# Factors influencing the work efficiency of district health managers in low-resource settings: a qualitative study in Ghana

**DOI:** 10.1186/s12913-016-1271-3

**Published:** 2016-01-14

**Authors:** Marc Bonenberger, Moses Aikins, Patricia Akweongo, Kaspar Wyss

**Affiliations:** 1 Swiss Centre for International Health, Swiss Tropical and Public Health Institute, Socinstrasse 57, Basel, 4002 Switzerland; 2 University of Basel, Basel, Switzerland; 3 School of Public Health, University of Ghana, Legon, Ghana

**Keywords:** District health managers, Management, Efficiency, Health system, Decentralisation, Decision space, Ghana, Sub-Saharan Africa

## Abstract

**Background:**

There is increasing evidence that good district management practices can improve health system performance and conversely, that poor and inefficient management practices have detrimental effects. The aim of the present study was to identify factors contributing to inefficient management practices of district health managers and ways to improve their overall efficiency.

**Methods:**

Nineteen semi-structured interviews were conducted with district health managers in three districts of the Eastern Region in Ghana. The 19 interviews conducted comprised 90 % of the managerial workforce in these districts in 2013. A thematic analysis was carried out using the WHO’s leadership and management strengthening framework to structure the results.

**Results:**

Key factors for inefficient district health management practices were identified to be: human resource shortages, inadequate planning and communication skills, financial constraints, and a narrow decision space that constrains the authority of district health managers and their ability to influence decision-making. Strategies that may improve managerial efficiency at both an individual and organizational level included improvements to planning, communication, and time management skills, and ensuring the timely release of district funds.

**Conclusions:**

Filling District Health Management Team vacancies, developing leadership and management skills of district health managers, ensuring a better flow of district funds, and delegating more authority to the districts seems to be a promising intervention package, which may result in better and more efficient management practices and stronger health system performance.

**Electronic supplementary material:**

The online version of this article (doi:10.1186/s12913-016-1271-3) contains supplementary material, which is available to authorized users.

## Background

There is increasing evidence that good management practices can improve health system performance [1]. According to the World Health Organization (WHO) [2] managers conduct good management practices when they “provide direction to and gain commitment from partners and staff, facilitate change and achieve better health services through efficient, creative and responsible deployment of people and other resources”. Studies indicate that good management practices are associated with lower patient mortality, higher institutional income, greater levels of patient satisfaction, and thus higher overall performance [1, 3, 4].

As in other district health systems in low- and middle-income countries (LMICs), district health managers in Ghana form the link between the strategic levels (national and regional levels) and operational levels (district and sub-district levels), and are responsible for managing all areas of health service delivery at the district and sub-district levels [5]. They are organised into District Health Management Teams (DHMTs) and members of the teams are composed of administrative, technical and operational managers. The work places of DHMTs are the District Health Administrations (DHAs), which are located in every district in Ghana. DHAs report to, and are supervised by, their respective Regional Health Administration (RHA), which is in turn accountable to the central administration at national level.

In countries which have implemented health sector decentralisation policies, district health managers often have a so-called broadened “decision space” [6], which refers to the effective range of choice within the various functions of the health system such as financing, service delivery, human resources (HR), and governance. In Ghana, decentralisation was initiated following the Ghana Health Service (GHS) and Teaching Hospital Act (Act 525) in 1996, which involved de-concentration of authority to the RHAs and DHAs in the country [6, 7]. Benefits of decentralisation identified in various countries include higher regional and local authority, accountability, improved implementation of health care strategies based on need, greater efficiency, and increased responsiveness to community requirements [8–11]. However, health care decentralisation has also been associated with negative effects, such as the delayed transfer of funds from national government, lack of technical capacity of local governments, and inequity [12–15].

**Fig. 1:**
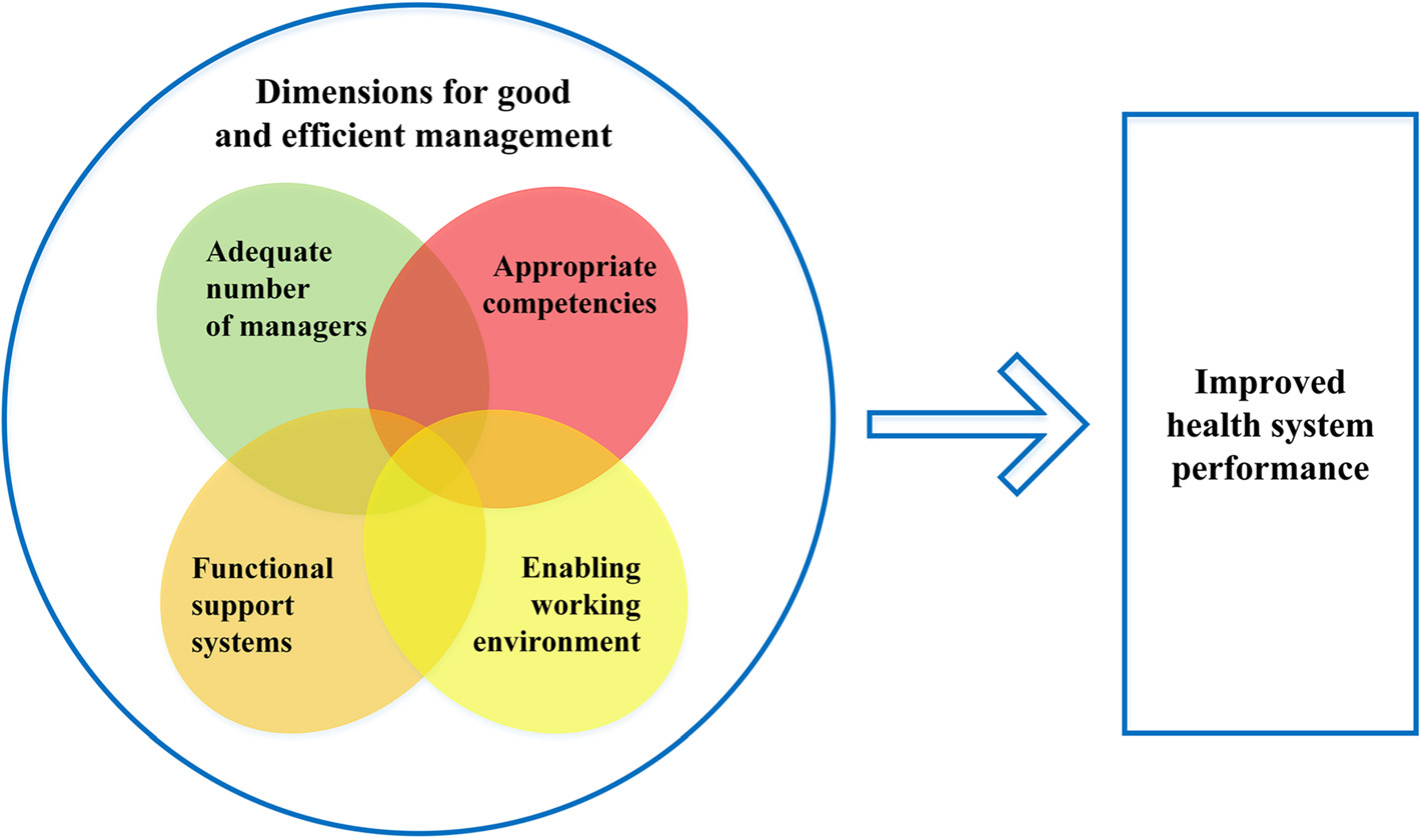
Leadership and management strengthening framework. Adapted from WHO [16]

The WHO’s [16] leadership and management strengthening framework proposes that for good and efficient management at the operational level, there has to be a balance between four dimensions. First, there needs to be an adequate number of trained managers. Second, managers need to have appropriate competencies, such as knowledge, skills, attitudes and behaviours. Third, critical support systems must be functional and accessible to managers. Such support systems include planning, financial management, information system for decision-making, human resource management (HRM), and management of stocks and assets (e.g. drugs, buildings, vehicles, and equipment). Fourth, there must be an enabling work environment with regards to roles and responsibilities, supervision and incentives, organisational context and rules, as well as the broad cultural, political and economic context. The framework suggests that strengthening these dimensions can result in more effective health systems and services through an improved and more efficient management (Fig. 1).

This study was carried out within the framework of PERFORM, a HRM intervention program, which aims at identifying ways of strengthening decentralized district management to improve health workforce performance in sub-Saharan Africa [17]. The aim of the present study was to identify factors contributing to inefficient management practices of district health managers, coping strategies to alleviate such factors, and ways to improve overall efficiency of district level managers.

## Methods

### Study setting

The study was conducted in the Eastern Region of Ghana in the Akwapim North, Upper Manya Krobo and Kwahu West districts. These districts were selected by the PERFORM country research team in close consultation with the regional authorities of the Ghana Health Service on the basis of their performance, and interest to take part. Performance of the districts was rated based on information from the national health league table according to which the 16 districts of the region were classified into good, moderate and poor performers. Eleven districts expressed an interest to participate. The final selection was for Kwahu West (well performing), Akwapim North (moderately performing), and Upper Manya Krobo (poorly performing) [18]. While Akwapim North and Kwahu West can be classified as rural and semi-urban respectively, Upper Manya Krobo is predominantly rural and belongs to one of the least developed districts in the region. It is with this context in mind that its classification as a poorly performing district should be understood [19].

At the time of the study in 2013, there were 21 DHMT members working in the districts of which eight were in Akwapim North, seven in Upper Manya Krobo, and six in Kwahu West. According to baseline data collected for PERFORM, the DHMTs were responsible for 22 health centres and 37 Community-based Health Planning and Services (CHPS) facilities at sub-district level, with the highest number of health facilities located in Kwahu West. Although all districts have a hospital, these have their own administrations and are thus not managed by DHMTs. The total population living in the catchment areas of the health facilities in the three study districts summed up to 317,989 people. Akwapim North had with 142,275 people the greatest catchment population and Upper Manya Krobo with 78,158 people the smallest. All three DHMTs managed a total number of 291 health workers of which the DHMT in Akwapim North managed with 140 workers (48.1 %) the highest number. Community Health Nurses (CHN) at the district level form the largest health workforce and the administrative workforce constitutes the lowest workforce. Due to the low numbers of trained doctors, doctors in Ghana usually are not found at sub-district level and hence are substituted by medical assistants (Table 1).

**Table 1:** Key characteristics of the three study districts in Ghana

		District		
	Total	Akwapim North	Upper Manya Krobo	Kwahu West
Number of district health managers	21	8	7	6
Health services	59	22	11	26
Health centres	22 (37.3 %)	9 (40.9 %)	5 (45.5 %)	8 (30.8 %)
CHPS facilities	37 (62.7 %)	13 (59.1 %)	6 (54.5 %)	18 (69.2 %)
Health workers^a^	291	140	72	79
Medical assistants	8 (2.7 %)	5 (3.6 %)	2 (2.8 %)	1 (1.3 %)
Midwives	35 (12.2 %)	22 (15.7 %)	4 (5.6 %)	9 (11.4 %)
Auxiliary nurses	21 (7.2 %)	11 (7.9 %)	5 (6.9 %)	5 (6.3 %)
Community health nurses	152 (52.2 %)	48 (34.3 %)	52 (72.2 %)	52 (65.8 %)
Health care assistants	9 (3.1 %)	8 (5.7 %)	1 (1.4 %)	0 (0 %)
Technical staff	23 (7.9 %)	12 (8.6 %)	4 (5.6 %)	7 (8.9 %)
Administrative staff	1 (0.3 %)	1 (0.7 %)	0 (0 %)	0 (0 %)
Orderlies	35 (12.0 %)	27 (19.3 %)	3 (4.2 %)	5 (6.3 %)
Support workers	7 (2.4 %)	6 (4.3 %)	1 (1.4 %)	0 (0 %)
Population in catchment areas	317,989	142,275	78,158	97,556

^a^In Ghana, other essential professions such as medical doctors and enrolled nurses usually do not work in sub-district facilities and are thus not represented in the statistics

*CHPS*: Community-based health planning and services

CHPS is a primary health care programme in Ghana, which was pilot-tested in the early 1990s and then rolled-out as a national policy from the late 1990s onwards in order to increase health service accessibility of people living in rural and remote areas [20, 21]. CHPS facilities are managed by CHNs who provide mobile doorstep health services to community residents.

### Data collection

All district health managers working in the study districts were asked to take part in the study. A semi-structured guide was developed and used for the interviews [see Additional file 1]. The guide was divided into the following thematic areas: usual work activities, causes of inefficient district health management practices, strategies to cope with such factors, and possible ways to improve efficiency. Efficiency was thereby defined as the ability to produce management outputs by making an optimal use of resources, including time [22]. All interviews were conducted by the lead author from August to September 2013. Respondents were contacted by phone and the study explained. If the respondents were willing to take part an appointment was scheduled. The interviews were carried out in the offices of the respective managers. All interviews were conducted in English and taped with a digital audio recorder. In total, 19 interviews were conducted with district health managers comprising 90 % of the managerial workforce of the study districts at the time of the data collection in 2013.

### Data analysis

All interview tapes were transcribed verbatim and a thematic framework method analysis conducted [23]. We used QSR NVivo version 10 for the analysis. The transcripts were repeatedly read in order to familiarise with the data. The four dimensions from the WHO leadership and management strengthening framework [16] were used as the main themes of the analytical framework, each being subdivided into the categories “constraints”, “coping strategies”, and “measures to improve efficiency”. Sub-categories were identified in the transcripts through the use of codes as a way of labelling important passages. Interrelated or similar codes were grouped together into different sub-categories. The analytical framework was applied by indexing all transcripts using themes, categories and identified sub-categories. Contents of categories and its sub-categories were then summarised in analytical memos.

### Ethical considerations

As PERFORM is coordinated by the Liverpool School of Tropical Medicine (LSTM), ethical clearance for the whole study was obtained from the Research Ethics Committee of LSTM (ID No.: 12.09). For the present study additional ethical clearance was obtained from the Ghana Health Service Ethical Review Committee (ID No.: GHS-ERC: 13/05/12). Before the start of the data collection we received written clearance from the Eastern Regional Health Administration, Koforidua, Ghana. Participants gave verbal informed consent to participate in the study, for which no particular written consent form was necessary, as they already had provided a general consent for PERFORM. All personal data were anonymised prior to the analysis.

## Results

### Adequate number of managers

As shown in Table 2, all DHAs had vacancies of essential DHMT personnel. Nine positions were not filled at the time of the study. According to this distribution, a health services administrator and a health promotion officer were available in only one district, and a nutrition officer and a supply officer in two districts. None of the study districts had a HR officer. Respondents emphasised that the DHAs had repeatedly sent letters to the RHA of the Eastern Region requesting for staff to fill their vacancies. By the end of the data collection period, the RHA had not been in the position to fill any of the DHMT staff vacancies in the districts. This was likely a result of an embargo of new employments for all non-clinical governmental health workers that was introduced by the Government of Ghana in 2010 in order to reduce the burden of salary costs within overall governmental expenditures [24, 25].

**Table 2:** Availability of DHMT staff in the study districts and vacancies

Profession	Akwapim North (*n* = 8)	Upper Manya Krobo (*n* = 7)	Kwahu West (*n* = 6)	Vacancies (*n* = 9)
Administrative managers	2	1	1	2
District directors of health services	1	1	1	0
Administrators	1	0	0	2
Technical managers	4	4	4	3
Public health nurses	1	1	1	0
Disease control officers	1	1	1	0
Health information officers	1	1	1	0
Nutrition officers	0	1	1	1
Health promotion officers	1	0	0	2
Operational managers	2	2	1	4
Finance officers	1	1	1	0
Supply officers	1	1	0	1
Human resource officers	0	0	0	3

Due to these HR shortages district health managers were required to take over additional tasks on top of their own duties, which represented a major constraint for most of the respondents, particularly because many of these tasks such as buying, managing and issuing drugs, where highly time consuming, thereby increasing the already high workload. Moreover, many respondents emphasised that staff capacity in each DHA department was insufficient, with only one person available per job category in most cases. District health managers often stated, that the presence of a colleague and/or assistant would be essential for completing their work tasks in time.

*“I am the only one person around [in the department]. I am the only one doing basically everything. One person does everything is like one man thousand. I do the fieldwork and I do the administrative work. I think I need a helping hand”* **(DHM #14).**

*“We used to be two, I used to have a boss to work with. […] But for now I’m alone, so I have to take care of the EPI, and to take care of the surveillance”* **(DHM #17).**

As delays were often a result of staff shortages, many of the described coping strategies also relate to HR. As usually not all DHMT members are invited to attend meetings and workshops at regional level, those who stay in the district often take over responsibilities of managers that have to attend. Task sharing was generally a coping strategy for time-bound activities so that these could be completed in time.

*“At the DHA, because we are limited, the staffs are not adequate. And because we are not enough, jobs are shared among us. Like somebody can do some part of it for you so that you also do other things. So whatever schedule you are put on you make sure you do it and do it well”* **(DHM #18).**

Executive managers, commonly district directors of health services and public health nurses, also reported that they delegate some of their work tasks to subordinate DHMT members when they were unable to complete them.

Those respondents that performed double-functions due to HR shortages also stated that they prioritise their core duties, thereby neglecting additional duties.

*“Last week, because of the measles immunisation programme that we had, I couldn’t finish my monthly report [of routine health data]. So this week I have to finish with it. Meanwhile, we had an email that we have to update our staff list. By Monday we should submit it, but I have to forgo that one and make sure I finish with my core duty, which is the monthly report, and then tackle the other one later”* **(DHM #16).**

All DHAs made use of national service personnel to fill their vacancies. These are personnel who complete a mandatory one year service to the country after graduating from accredited tertiary institutions [26]. It was reported that during the time of the interviews national service personnel were carrying out work tasks in HR, supply, disease control, and were also used as accountants. A similar strategy was to assign non-DHMT staff with tasks of vacant DHMT positions, such as secretaries taking over HR functions or those of health services administrators. One district health manager also reported to have trained a community health nurse from the sub-districts, who was now able to assist in data reporting and other activities.

In order to alleviate HR constraints, the respondents frequently highlighted the need for the higher levels to ensure the availability of adequate human resources in all DHAs so that essential DHMT positions, such as HR officers, supply officers and health services administrators do not remain vacant and that also a sufficient number of assistants would be available in each department. It was argued that this would likely result in efficiency gains through a reduction of workload allowing DHMT members to focus on their core duties.

*“We should have enough staff. That’s what I think should be, but that’s not the case. You have to double up, do this, do that, a whole lot. If you are the health information officer your focus should be on health information, if you are the HR officer your focus should be on HR. If we had fixed personnel handling those things, the work would be a bit smooth for us”* **(DHM #16).**

### Appropriate competencies

Many district health managers criticised that because they were taking over tasks of vacant professional positions, the quality of work outputs was affected, as they were carrying out tasks for which they were not trained for and for which they lacked the necessary skills and experience. For instance, in one district the health information officer was also responsible for HR management, including maintaining the HR database, organising in- and out-transfers and promotions, and counselling of sub-district staff, although the person had not received training in these areas whatsoever. The concerned DHMT member stressed that this lack of training in HR management resulted in mistakes, thereby affecting efficiency.

*“When we have new staff we have to send their details to Accra for input. When they are being promoted you have to send their details to Accra for it to be inputted so that they get their salary. And because it is not my core duty, I do my best, but at times there are some corrections so that I have to come back [to the office] and print and all those things […]. So I have to travel back and forth until I get it right”* **(DHM #16).**

In addition, insufficient planning, communication, and time management skills were also frequently reported as the cause of inefficient management practices. Although DHMTs develop annual action plans at the beginning of each year, district health managers reported difficulties in working according to these and to translate plans into weekly DHMT activities. Therefore, in order to achieve workflow gains at the district level it was stressed that DHMTs should improve their planning skills, put a stronger emphasis on weekly planning, and develop individual work plans that are consistent with those of the DHA.

*“If we are able to work according to action plans it will help improve efficiency, because if you are able to look at action planning, you know that this is what we have for this week. Then you can also plan your own activities in line with that. It will help”* **(DHM #12).**

However, the importance of sharing individual plans during weekly DHMT meetings was also emphasised so that everyone in the DHA, including non-DHMT staffs, were aware of what colleagues had planned for the week. Respondents argued that this would likely lead to improved collaboration and to a reduction of duplicated activities resulting from poor communication.

Respondents also stressed that efficiency gains could be made by becoming more time conscious, as this would likely result in the reduction of time spent on unproductive activities, such as having lengthy private conversations with colleagues, sitting idly in the office, having extended breaks, and engaging in private activities during duty hours.

### Functional support systems

The efficiency of district health managers is dependent in part on how well critical support systems function. District managers in Ghana have access to a broad range of management systems, most importantly planning, finance, information, HR, and procurement and distribution systems for drugs and other commodities. Responses centred mainly on financial constraints, but problems regarding other management systems such as HR, monitoring and transport were also mentioned as consequences of these constraints. Although it can be expected that problems regarding other management support systems do also exist [16], these were not mentioned by any of the respondents and thus might not be regarded as major constraints.

Financial problems of the district and sub-district levels mainly result from the government not releasing funds in time. These “cash flow” problems led to the inability to do regular maintenance of vehicles and technical equipment, buy fuel, and ensure the supply of essential office materials. Such problems, in turn, were main barriers preventing district health managers to implement planned activities in time and thus contributed substantially to inefficiencies. It was emphasised that regularly the only funds available were those made available for vertical programme funds – such as for the Expanded Programme on Immunisation (EPI) – or other donors. There was however no flexibility in using these resources as they were earmarked for specific programmes.

*“There are financial constraints here, because the government is not releasing funds to the DHAs. Formerly we were being given the SBS (Sector Budget Support) and GoG (Government of Ghana) funds. But for some time now we have not been receiving them. Only programme funds, but you cannot use them for other things. So there are financial constraints, very serious ones. Now it is not only here. The problem is all over”* **(DHM #19)**.

*“We have severe funding constraints. We are not able to do monitoring and other facilitative supportive visits that we want to do. For the whole year we have done monitoring and supervision only in the first quarter. In the second quarter we could not do it. And this quarter we are also not able to do it, because of a lack of funds for fuel to travel outside. The vehicle is there, but funds for fuel are not”* **(DHM #5)**.

As funds were generally limited in the DHAs, district health managers reported making use of alternative funding sources to carry out planned activities. For instance, in contrast to governmental health funds, respondents stressed that funds for vertical programmes and from donors were usually released in time. As the frequent inability to buy fuel prevented regular sub-district visits for monitoring and supervision purposes, district managers reported to use the opportunity to conduct such activities when facilities were visited in the frame of vertical programme or donor activities. However, such monitoring and supervision visits can be regarded as spot checks rather than a systematic visit of all facilities in the district.

Under rare circumstances, such as when money was urgently needed to attend important national meetings or conferences, internally generated funds (IGFs) – revenues generated through the activities of sub-district health facilities – were also used. In addition, district health managers reported that they sometimes used their private money to fund urgent activities, and were paid back by the DHAs once money became available.

*“What we have been doing here is that at times we pre-finance some programmes. Like if you want to do supportive supervision and we see that it is very important for us to do that to correct some things immediately, if there is no fuel in the vehicle we normally buy by ourselves and then bring the receipt so that when there is money then they refund it”* **(DHM #2).**

When funds become available, it was reported that these were often not sufficient to conduct all activities as planned, such as visiting all sub-district facilities for monitoring and supervision. In such instances, a certain number of health facilities are selected purposefully, for example only health centres and CHPS facilities in one or two sub-districts. The remaining facilities in other sub-districts are then visited at a later stage when the opportunity arises.

Most district health managers stressed that the national level must ensure an adequate flow of public health funds so that health activities could be carried out in time, thereby improving DHMT efficiency.

*“I think that basically it is funds. I think if we had the funds we could do things at the right time, but because we don’t have funds some of the things are just there. We cannot do anything”* **(DHM #9).**

*“If the [financial] resources would come on time for you to work with, it would help us. But the funds are not coming on time and then when it comes we have limited time to finish with it”* **(DHM #18).**

### Enabling environment

Factors critical for good and efficient management include policies, legislation, norms and standards, which support the appropriate delegation of authority; adequate support for managers, especially regarding access to information, communication, and supervision; financial and non-financial incentives for good management; and accountability [27]. Despite this broad range of possible topics concerning the work environment, district health managers primarily regarded inadequate planning and communication of the higher health system levels as a constraint for efficient district management.

Because decisions of the higher levels generally supersede those of the district level, demands from these levels have higher priorities than all activities routinely conducted by district health managers. A high share of the activities conducted by district health managers is usually required by the national and regional administrations leaving little room for them to focus on district specific activities.

Most respondents complained that the higher levels regularly informed them about upcoming activities at very short notice so that these activities are difficult to incorporate in their work schedules. As such, they interrupted their plans and resulted in implementation delays.

*“What happens is that, if nothing comes from the region, if no programmes come from the regional level, whatever I have planned I will do it within the week. I’m able to do it”* **(DHM #17).**

It was emphasised that particularly the RHA often sent invitations for meetings and workshops at very short notice, although respondents admitted that information practices of the RHA had recently improved. In addition, district health managers complained that workshops were often held over several days with participation being mandatory. Such workshops frequently interfered with time-bound activities at district level.

*“You know, you plan your week. You know this week you are going to do ABCD. And then a letter will come from the region, you have one week workshop, you have to stop whatever you are doing and then go”* **(DHM #11).**

Due to the frequent interruptions through unplanned activities time-bound activities were regularly at risk of not being completed in time. As a result, almost all district health managers stated that they were usually working overtime, and sometimes also on weekends in order to meet their deadlines. Some respondents also reported that they were regularly working in the evening hours after workshops so as to complete activities in time.

Many district health managers stressed that the higher levels should improve their planning and communication concerning the district level, which particularly referred to meetings and workshops, but generally to all activities that directly affected the districts. Although national annual plans exist and were widely distributed within the GHS, it was emphasised that these plans only included national celebrations, health weeks and some management meetings. District health managers, therefore, frequently stressed that both the national and the regional levels should inform regularly and more timely on all upcoming activities so that DHMTs could incorporate these in their work plans.

*“So they should give us a work plan: these are the meetings and workshops we are going to hold and this is the time we will be holding them, these are the personnel that will be involved […]. But they let us do our plans and they also do their own and they are interfering left and right. They are our biggest problem”* **(DHM #15).**

In order to ensure that meetings and workshops did not interfere so frequently with activities at district level it was recommended that the higher levels should better coordinate such events, for instance by involving the DHAs in the planning process or at least by considering district work plans.

## Discussion

Our findings indicate that there are limitations in all four dimensions of the WHO’s leadership and management strengthening framework. All study DHAs had HR shortages, as essential DHMT positions such as HR officers and health services administrators were vacant and thus the number of district health managers was inadequate. Our findings also suggest that planning as well as communication of district managers are both inadequate and, therefore, managers lack appropriate competencies in these areas. That several of the critical support systems, especially the finance management system, are inadequate is a further, major finding of this study. Also the narrow decision-space of district health managers in Ghana does not seem to provide an enabling working environment to solve district specific problems.

DHMT members foremost identified difficulties in financial management processes as a determinant element. Financial constraints are indeed well-known to many health systems in LMICs [28] and were identified as main barriers to efficient district health management also in this study. Abekah-Nkrumah et al. [29] have shown in a review of the budgetary process in the Ghanaian health sector that although channels for disbursing administrative funds are smooth, disbursements are erratic due to challenges with cash flow. In addition, cumbersome central procedures for approval of local budgets and a lack of administrative capacity at the national level regularly result in delayed approvals of district funds [29, 30]. That this untimely release of administrative funds disrupts the implementation of DHMT activities is also a major finding in another study from Ghana [13]. Besides financial constraints, HR shortages in the DHAs, inadequate planning skills of district health managers, and poor planning and communication of upcoming activities from the higher levels are also identified as key factors for inefficient district management practices. These findings are supported by quantitative data we reported in a previous article on DHMT time use practices, which showed that great shares of managers’ capacities are used for only few district activities by at the same time neglecting other important activities, which we attributed mainly to HR shortages in the DHAs as well as insufficient planning and coordination by the higher health system levels [18].

District health managers reported that DHMTs spend time on unproductive activities such as having lengthy private conversations with colleagues and extended breaks. Although managers attributed these activities to fellow DHMT members and never to themselves, such statements are at odds with the frequent requirement to work overtime and on weekends due to high workloads. This, however, indicates that DHMT members may not use their work time efficiently and that gains can be achieved without additional resources through proper time management and training.

Several factors affecting efficiency as proposed by the WHO’s leadership and management strengthening framework [16] were not identified by respondents. Such factors include inadequate access to supportive supervision and appraisals, HR management systems, collaboration with other stakeholders at district level, and incentives for good performance. Although district health managers in the study districts may not perceive these factors as primarily constraining their efficiency, research conducted in other LMICs suggests that such factors may indeed affect managerial efficiency [27, 31].

Common strategies of district health managers to cope with the untimely release of funds are to carry out routine activities while on duty for vertical programs or donors, to borrow IGFs from sub-district health facilities, or to use private cash to advance funds for DHMT activities. Such strategies confirm those reported in a study conducted by Asante et al. [13] in Ghana. Regarding the other strategies described in this study, some are themselves a source of inefficient district management. For instance, while prioritising core duties increases efficiency of such activities, additional duties are neglected at the same time, thereby affecting efficiency. Also making use of unqualified staff to carry out DHMT work tasks may improve efficiency in some work areas due to the greater number of workers, but may simultaneously decrease efficiency in other areas through mistakes resulting from a lack of appropriate training and skills. Moreover, using unqualified staff to carry out qualified work is also likely to have negative effects on the quality of work outputs.

It is generally assumed that managers are more efficient in decentralised health systems, given that they have better control over resources and activities and are in a better position to plan and prioritise as compared to managers in centrally managed health systems [22, 32]. Very limited financial resources are generated at district level and these can be administered by district managers. However, most of these resources are made available to local managers by the national and the regional administration [6, 19] and here the district managers have only little control. In addition, studies also emphasised the low decision-making authority of Ghana’s district health managers [5, 6, 33], which was attributed to the colonial organisation structure inherent in the modern health system encouraging a centralised hierarchical administration culture with little involvement of the district level in planning and implementation of health services [34]. In our study, we identify both issues – low control over resources coupled with little authority over district activities – as major factors for inefficient district management. Our findings thus confirm the rather narrow decision space attributed to district health managers by studies carried out in Ghana in recent years [5, 6].

This study has limitations. As the focus is only on district health managers in three districts, perceptions concerning these districts may not be generalizable for all areas in the country. However, given their broader administrative responsibilities, findings regarding the higher health system levels are likely to also prevail in other areas. Moreover, the issues emerging from the data reveal relationships significant for management strengthening in LMICs and are thus relevant for researchers, donors and policy makers. Although the three DHAs included in this study differ in terms of factors such as DHMT composition, distance to the regional and national administrations, and socio-economic status of the districts, we pooled all data and, by doing so, may have masked discrepancies between the districts that could have been revealed through a stratified analysis. We also recognise the limitation of not having explored the perceptions from regional and national health managers, health workers as well as from patients and health service users concerning DHMT efficiency. Such triangulation might have added different perspectives.

## Conclusions

This study has shown that inefficient district health management is mainly a result of HR shortages at the DHAs, inadequate planning and communication skills of district health managers, financial constraints, and a narrow decision space giving district managers little authority over activities. The findings thus suggest limitations in all four dimensions of the WHO’s leadership and management strengthening framework. As these are closely interrelated, efforts to strengthen management at district level are unlikely to succeed when these are not tackled at the same time. It is, therefore, not sufficient to just carry out interventions at the district level by improving management skills of district health managers or increasing their number. Although developing management skills of district health managers is undoubtedly important, our findings strongly suggest that the wider health system must also be considered in order to achieve better management efficiency. Filling vacancies of DHMT members in all districts, developing leadership and management skills of district health managers, ensuring a better flow of district funds, and delegating more authority to the districts seems to be a promising intervention package, which may result in improved and more efficient management practices and stronger health system performance.

## Additional file

Additional file 1:
**Interview guide.** (DOCX 35 kb)

## Abbreviations

CHN: Community health nurse
CHPS: Community-based Health Planning and Services
DDHS: District director of health services
DHA: District Health Administration
DHM: District health manager
DHMT: District health management team
EPI: Expanded Programme on Immunisation
GHS: Ghana Health Service
HR: Human resources
HRM: Human resource management
HS: Health system
IGF: Internally generated funds
LMICs: Low- and middle-income countries
RHA: Regional Health Administration
WHO: World Health Organisation
