# Supplementary material for: Factors influencing the work efficiency of district health managers in low-resource settings: a qualitative study in Ghana
Source: BMC Health Serv Res. 2016 Jan 14;16:12. doi: 10.1186/s12913-016-1271-3 (PMC4712591; doi:10.1186/s12913-016-1271-3)
Supplement: Additional file 1: — Interview guide. (DOCX 35 kb) [file 12913_2016_1271_MOESM1_ESM.docx]

**Interview guide for DHMT efficiency study**

**Usual work activities**

1. Please describe your main work tasks in your job as a _____ (state job title)?
2. Which of these work tasks, in your experience, are most time consuming?
3. Are you aware of your job description as a _____ (state job title)? If so, please shortly describe the written tasks.
4. What are the main work tasks you usually perform, which are not listed in your job description?
5. Could you please estimate how much of your activities during a usual workday are planned and how much unplanned?
6. What are common unplanned activities?

**Causes of inefficient DHM practices**

1. How do your unplanned activities interact with your normal work schedule?
2. What are usual main constraints when you realise that you cannot complete your activities during the week as intended?
3. To which activities you think you allocate too much time and to which you consider not devoting enough time?

**Coping strategies**

1. When you realise that you are not able to complete all activities during the week as intended, to which type of your activities you usually give priority and which tend not to be implemented?
2. How do you usually decide which of your activities are more important than others?

**Improvement of efficiency**

1. When you think of the constraints in regard to your time-allocation, what do you think you could change to improve your efficiency?
2. What are constraints you think you cannot change, and why?
3. What do you think should be improved so that you can allocate more time to activities that are important and less to those that are not so important?
4. Do you have suggestions what the Ghana Health Service should change in order to remove the biggest constraints, which you cannot solve by yourself?
5. When you think of the current performance of the whole DHMT and the district health service, how, in your opinion, can performance be improved in regard to efficient time allocation?
